# Denoising Ocean Turbulence Microstructure Signals for Application in Estimating Turbulence Kinetic Energy Dissipation Rates Based on EMD-PCA

**DOI:** 10.3390/s22124413

**Published:** 2022-06-10

**Authors:** Xue Chen, Xiangbin Zhao, Yongquan Liang, Xin Luan

**Affiliations:** 1 College of Computer Science and Engineering, Shandong University of Science and Technology, Qingdao 266590, China; lyq@sdust.edu.cn; 2 Department of Information Engineering, Shandong Foreign Trade Vocational College, Qingdao 266100, China; zhaoxiangbin0328@163.com; 3 College of Information Science and Engineering, Ocean University of China, Qingdao 266100, China; ouc_xluan@163.com

**Keywords:** ocean turbulence, noise, empirical mode composition, principle component analysis

## Abstract

When ocean turbulence signals are collected using turbulence observation instruments in real marine environments, the effective signals in the acquired data set are often polluted by noise. In order to eliminate the noise component contained in the non-stationary and nonlinear ocean turbulence signals, a new multi-scale turbulence signal denoising method is proposed by combining the empirical mode decomposition (EMD) and principle component analysis (PCA). First, the time series of turbulence signals are decomposed into a couple of components by EMD algorithm and approximately calculate the noise energy in each intrinsic mode function (IMF). Then, PCA is implemented on each IMF. The appropriate principal components are selected according to the decomposition characteristics of PCA and the noise energy proportion in IMF. Each IMF is reconstructed by the selected principle components. At last, the effective ocean turbulence signals are reconstructed by the corrected IMFs and the residue. Ocean turbulence signals collected in the South China Sea (SCS) are used to evaluate the effectiveness of the proposed method. The results show that the proposed method can effectively eliminate the noise and maintain the characteristics of the effective turbulence signals under high noise. Turbulence kinetic energy (TKE) is also estimated from the denoised signals, which provide a reliable data basis for the analysis of the turbulent characteristics in later stage.

## 1. Introduction

Ocean turbulence mixing is one of the most challenging research fields in physical oceanography as it is unsteady and irregular. As one of the main physical processes, turbulence controls the ocean overturning circulation at a wide range of temporal and spatial scales because of its influences on climate. At small scales, turbulence affects the ocean circulation and ecosystems by enhancing the transport of heat and nutrients within the water column. Therefore, the study of ocean turbulence can not only improve people’s understanding of energy exchange in the marine environment and global climate change, but also strengthen the prediction system of marine climate and disasters [1,2,3].

Highly accurate turbulence data is the basis to analyze ocean turbulence characteristics. Oceanic turbulence measurements have been taken for more than 50 years since the pioneering work by Grant in 1962 [4]. In the open ocean, turbulence is usually assessed by measuring velocity fluctuations at dissipation scales using shear probes, while temperature fluctuations are measured with thermistors or platinum film thermometers. By mounting on different ocean observation platforms, the shear probe can continuously achieve abundant turbulence data, which provides rich data resources for turbulence characteristic analysis. However, to measure a few centimeters of turbulence microstructure in the open sea is quite difficult because it is inherently chaotic, unsteady and unpredictable as well as being a tough experiment environment. The collected turbulence data is inevitably contaminated by various noise, such as vibration noise of the observation instrument and cable tension, the high frequency electronic system noise, and natural noise (sea wind and waves in the water column or the collision of the probes [5,6]. These noises will affect the evaluation of ocean turbulence characteristics, such as the dissipation rate of turbulent kinetic energy (TKE). So, the noise should be removed from the measured turbulence signals and turbulence signal denoising is an important segment to acquire high-precision data.

In order to eliminate the noise component in the ocean turbulence signal correctly and effectively, researchers have proposed different methods [7,8,9]. In the past several decades, the structure and accuracy of the measuring sensors have continuously improved, and the sensor technology has been perfected. Researchers then tried to find an effective denoising method at the algorithm level. Traditional denoising methods, such as low-pass filter based on Fourier transform, the spatial power-response transfer function and wavelet transform denoising algorithm, are the most common denoising methods. Each denoising algorithm has its own advantages and disadvantages. The low-pass filtering algorithm can filter out high-frequency signals by setting the cut-off frequency, which is simple and easy to use. However, this algorithm easily filters out the effective information in the high-frequency part and has no denoising effect on removing the vibration noise distributed in the low frequency. The wavelet transform denoising algorithm requires selection of the wavelet basis function and decomposition scale, which is not suitable for the non-stationary turbulence signals. The wavenumber spectrum calculated from the measured turbulence signals can be used as one of the important standards to evaluate its quality, so researchers try to eliminate the noise from the perspective of the characteristics of the signal itself. By installing the accelerometer sensor on the measuring instrument to measure the motion posture of the observation instrument, Lueck proposed the frequency response transfer function to correct the turbulence signals. By subtracting the coherent signals from the accelerometer, the shear probe signal vibration contamination is removed [5]. However, this method is only effective at reducing vibration noise in one direction. Goodman proposed a cross-spectrum denoising method to remove the noise caused by the instrument vibration [8]. Through calculating the weight value, all the coherent signals from the three-axis accelerometers can be obtained, and the vibration noise can be eliminated to some extent. However, this denoising method treats all the wavenumbers the same and lacks the optimal estimation of the weight value. Accurate and precise turbulence data are only achievable through the design of a suitable filter to process raw turbulence observed signals, and this, in turn, is of utmost importance since it dictates not only the fidelity of subsequent analysis on turbulence characteristics but also conclusions drawn from such analysis [9].

Recently, some methods for signal denoising scheme are introduced that are based on empirical mode decomposition (EMD) [10]. The EMD algorithm is a data-driven adaptive signal decomposition method, which can adaptively decompose the noisy signal into a set of intrinsic mode functions (IMFs) ordered by frequency. EMD was originally developed to process nonstationary and nonlinear data, such as experimental turbulence records [11,12]. Now, it has been further applied successfully to a variety of physical systems, such as earthquake, ocean, mechanical vibration analysis, sound processing and other fields of data processing and analysis. The basic principle of the denoising methods based on EMD are to reconstruct the signal with IMFs that directly remove the high-frequency part, previously filtered or thresholded. The direct removal method is easy to conduct but also removes the details of the signals, and the filtered or thresholded based on wavelet threshold is hard to determine the threshold of the method.

Following this idea, we present in this paper a denoising technique based on EMD and principal component analysis (PCA), which selects a few independent principal components from the original complex signal to represent most of the information of the original signal [13]. The turbulence denoising method based on EMD-PCA is designed to conduct in the frequency domain, which can achieve the optimal estimation of the observation spectrum and the dissipation rate of turbulence kinetic energy (TKE). Through decomposing the original turbulence signal into IMFs and estimating the noise energy using PCA algorithm, the appropriate principal components are selected for reconstruction. The sea data collected in the South China Sea with a moored turbulence measuring instrument (MTMI) is used to evaluate the validity of the improved turbulence denoising method. The outline of this paper is as follows. The turbulence observation instrument and the deployment are introduced in Section 2. Section 3 mainly presents the improved turbulence denoising method based on EMD-PCA in detail and gives the process to calculate the TKE dissipation rate. Sea data results to verify the validity of the improved denoising method are shown and discussed in Section 4, and conclusion remarks are given in Section 5.

## 2. Instrument and Deployment

The sea data used to verify the validity of the turbulence denoising method is collected in the South China Sea (21°09.900′ N 117°42.031′ E) with a moored turbulence measuring instrument (MTMI) designed by Ocean University of China (OUC). The MTMI is designed to collect abundant turbulence microstructure time series from the deep sea for a long time at a fixed level. In this experiment, the instrument was deployed in about 250-m-deep water, and it successfully collected turbulence microstructure time series for about 110 days.

**Figure 1:**
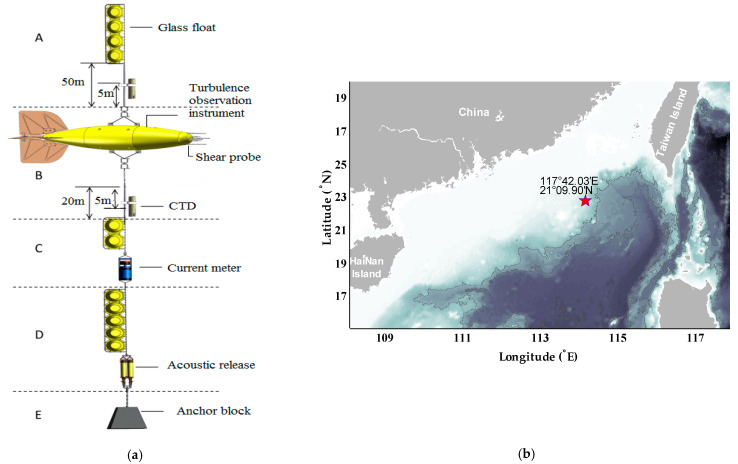
The self-designed MTMI. (**a**) The main structure of the MTMI; (**b**) the field test location of the whole observation platform in the South China Sea.

The main structure of the MTMI is shown in Figure 1. The core component of MTMI is the turbulent observing part (part B in Figure 1), which in the middle of the nose contains two orthogonal shear probes (PNS07) to measure the cross-stream velocity fluctuations. In the design, one of the shear probes is oriented to sense horizontal velocity fluctuations (*∂w*/*∂t*), and the other responds to the athwartships velocity fluctuations (*∂v*/*∂t*). To monitor the status of the instrument under the seawater, the three orthogonal accelerometers are housed in the instrument cabin by a righthand cartesian coordinate, which can provide information on three directions. All the voltage output of the sensors are digitized by 16-bit A/D conversion and finally stored in the storage card. Meanwhile, part C mounted a current meter to measure the current speed, direction and the ability of the instrument to respond to the ambient currents. Part A is mainly the glass floats designed to provide buoyancy and ensure the stability of the whole system. Part E is the anchor block. Under the effects of part A and part E, the whole system can maintain a vertical state underwater. To recover the whole system, the acoustic release in part D opens and releases the anchor block, then the whole system ascends to the sea surface under the effect of buoyancy. The structure of MTMI and the field test location of it in the South China Sea are shown in Figure 1. The asterisk in Figure 1b represents the field test location and the horizontal and vertical coordinates represent longitude and latitude, respectively.

## 3. Methodology

### 3.1. Multi-Scale Turbulence Signal Denoising Method Based on EMD-PCA

The EMD (empirical mode decomposition) method is a signal processing technique, which was originally developed to process nonstationary and nonlinear data, such as experimental turbulence records. Now, it has been further applied successfully to a variety of physical systems. The key benefit of using EMD is that it is automatic and fully data adaptive.

The EMD method decomposes the original signal S(t) into a finite number n of intrinsic mode functions (IMFs), which represent different time scales and frequency from high to low [11]:

(1) $$S(t)=\sum_{j=1}^{n}imf_{j}(t)+r_{n}(t)$$

IMFs can be expressed as ${\mathrm{IMF}}_{j}(t)=A_{j}(t)\cos\Phi_{j}(t)$, where $A_{j}(t)$ and $\Phi_{j}(t)$ represent the amplitude and phase of the j mode, respectively. Each IMF is characterized by a time-dependent $\omega_{j}(t)$, and a typical time scale can be obtained by averaging over the whole time interval. It can be seen that the essence of EMD decomposition is to decompose the characteristics of different scales in the signal. The signal is gradually decomposed to form a series of IMF components, and each IMF component reflects the intrinsic modal characteristics of the different scales.

Here, we analyze the signals in terms of energy. We use the EMD method to extract IMFs from a single mixed signal, which is a combination of a source signal and compound noises. The obtained IMF component can be defined as [14]:

(2) $$\varepsilon (imf_{j})=imf_{j}\cdot imf_{j}^{T}$$

Let $F_{j}=imf_{j}$ and $F_{j}=g_{j}+n_{j}$,where, $g_{j}$ is the effective signal component in $imf_{j}$ and $n_{j}$ is the noise component in $imf_{j}$. Then, the energy of each IMF component is recorded as $\varepsilon (F_{j}),\;\varepsilon (g_{j}),\;\varepsilon (n_{j})$. Based on the assumption that the effective signals have no correlation with the noise component, the denoising process can be considered as removing the noise energy $\varepsilon (n_{j})\;$ from the $\varepsilon (F_{j})$ [15].

In order to find uncorrelated dominant basis components, the PCA algorithm is used. By employing singular value decomposition, matrix U is referred to as a row basis representing the principal components of $Y_{L\times P}$. The singular values represent the standard deviations proportional to the amount of information contained in the corresponding principal components [16,17]. A reduced set of P basis vectors are selected from $U_{L\times P}$ using P first singular values.

According to the decomposition characteristics of the EMD method, $imf_{1}$ has the maximum amount of noise [10]. When using PCA to remove the noise component in $imf_{1}$, we only retain the first principal component, and the obtained component can be defined by $g_{1}=[u_{1}\;0\;0\cdots 0]P=u_{1}P_{1}$ and the noise removed can defined by $n_{1}=F_{1}-g_{1}=\sum_{i=2}^{N}u_{i}P_{i}$.

We record the energy of noise in $imf_{1}$ as $W_{1}$, which is calculated as [18,19]:

(3) $$W_{1}=\varepsilon (n_{1})={\left(n_{1}\right)}^{T}n_{1}={\left(\sum_{i=2}^{K}u_{i}P_{i}\right)}^{T}(\sum_{i=2}^{K}u_{i}P_{i})=(N-1)\sum_{i=2}^{K}\lambda_{i}$$

where, N represents the appropriate number of principal components. For $imf_{j}(j\geq 2)$, it is necessary to select an appropriate number of principal components for signal reconstruction. As each IMF component can be defined as $imf_{j}=F_{j}=g_{j}+n_{j}$, we transform each IMF into zero mean matrix according to PCA algorithm:

(4) $${\bar{F}}_{j}=F_{j}^{T}-E[F_{j}^{T}]=F_{j}-E[F_{j}]$$

${\bar{F}}_{j}$ has the same noise component as $imf_{j}$ and E[.] represents the mathematical expectation. After decomposition of the signal ${\bar{F}}_{j}$ using PCA, we can obtain the principal component that is expressed as $P_{1},\;P_{2},\cdots \;P_{K}$ and $\lambda_{1},\;\lambda_{2},\cdots \;\lambda_{K}$, respectively. Where $P_{i}$ and $\lambda_{i}$ represent as eigenvectors and eigenvalues of the covariance matrix, K is the length of $imf_{j}$. The first N principal components $P={\left[p_{1}^{T},p_{2}^{T},\cdots ,p_{N}^{T},0^{T},\cdots 0^{T}\right]}^{T}$ are selected, and reconstruction is conducted. The de-noising signal is then expressed as:

(5) $$g_{j}=[u_{1},u_{2},\cdots u_{K}]{\left[p_{1}^{T},p_{2}^{T},\cdots ,p_{N}^{T},0^{T},\cdots 0^{T}\right]}^{T}=\sum_{i=1}^{N}u_{i}p_{i}$$

The noise component removed from ${\bar{F}}_{j}$ can be defined as:

(6) $$n_{j}={\bar{F}}_{j}{-g}_{j}=\sum_{i=N+1}^{K}u_{i}p_{i}$$

According to the noise energy model, the energy of noise component in $imf_{j}(j\geq 2)$ can be approximately calculated according to $W_{1}$, which can be expressed as: $W_{j}=\frac{W_{1}}{\beta }\rho^{j}\;j\geq 2$, where, β≈0.719,ρ≈2.01 [20].

The energy of signal ${\bar{F}}_{j}$ and noise $n_{j}$ are expressed as $\varepsilon ({\bar{F}}_{j})\;$ and $\varepsilon (n_{j})\;$, respectively. Here, $\varepsilon ({\bar{F}}_{j})\;$ and $\varepsilon (n_{j})\;$ can be calculated as [21,22]:

(7) $$\varepsilon ({\bar{F}}_{j})={\left({\bar{F}}_{j}\right)}^{T}{\bar{F}}_{j}={\left(\sum_{i=1}^{N}u_{i}p_{i}\right)}^{T}(\sum_{i=1}^{N}u_{i}p_{i})=(N-1)\sum_{i=1}^{N}\lambda_{i}$$

(8) $$\varepsilon (n_{j})={\left(n_{j}\right)}^{T}n_{j}={\left(\sum_{i=N+1}^{K}u_{i}p_{i}\right)}^{T}(\sum_{i=N+1}^{K}u_{i}p_{i})=(N-1)\sum_{i=N+1}^{K}\lambda_{i}$$

Then, the signal to noise ratio can be obtained as follows:

(9) $$\frac{\varepsilon (n_{j})\;}{\varepsilon ({\bar{F}}_{j})\;}=\frac{\sum_{i=N+1}^{M}\lambda_{i}}{\sum_{i=1}^{M}\lambda_{i}}$$

If the appropriate number N of principal components is selected, the energy of removed noise $n_{j}$ will be the same as the noise energy contained in the original signal, that is $\varepsilon (n_{j})\;=W(imf_{j})$, namely that $\sum_{i=N+1}^{M}\lambda_{i}/\sum_{i=1}^{M}\lambda_{i}=W(imf_{j})/\varepsilon ({\bar{F}}_{j})$ is valid. It can be considered that the noise part of the signal ${\bar{F}}_{j}$ is completely eliminated. The number of principal components N should meet the following requirements:

(10) $$\sum_{i=N+1}^{M}\lambda_{i}/\sum_{i=1}^{M}\lambda_{i}\leq W(imf_{j})/\varepsilon ({\bar{F}}_{j})\leq \sum_{i=N}^{M}\lambda_{i}/\sum_{i=1}^{M}\lambda_{i}$$

Once the number of principal components N is determined, the reconstructed signal $g_{j}$ can be obtained.

### 3.2. Turbulence Signal Denoising Algorithm Based on EMD-PCA

Based on the above analysis, the specific steps of the proposed EMD denoising algorithm based on PCA are as follows:

Step 1: Preprocessing the observed original turbulent shear voltage signal and converting the voltage signal into physical turbulent shear signal x(t). Decompose the signal by EMD method and obtain the IMF components of each layer, which are recorded as $imf_{1}(t),imf_{2}(t)\cdots imf_{m}(t)$, as well as the remainder $r_{m}(t)$.

Step 2: Calculate the covariance matrix $C_{m\times m}$ as well as its eigenvalues $\lambda_{1}\geq \lambda_{2}\geq \cdots \geq \lambda_{m}$ and eigenvectors $U=[u_{1},u_{2},\cdots ,u_{m}]$ of each IMF component $imf_{j}$. Apply the PCA method on every $imf_{j}$ and obtain the principal component $P={\left[p_{1}^{T},p_{2}^{T},\cdots ,p_{m}^{T}\right]}^{T}$.

Step 3: For the first IMF layer component $imf_{1}$, let the number of principal components N=1 and reconstruct the signal based on the first principal component. The denoised signal is referred to as $g_{1}$. Calculate the noise energy $\varepsilon (n_{1})\;$, and estimate the noise energy for other IMF components $imf_{j}(j\geq 2)$.

Step 4: For other IMF components $imf_{j}(j\geq 2)$, choose the appropriate number of principal components to reconstruct the signal and get the denoised signal $g_{j}$.

Step 5: Cumulate and reconstruct the turbulence shear signal based on the $g_{j}(j\geq 1)$ of each layer and the remainder $r_{m}(t)$ The denoised turbulence shear signal $\hat{x}(t)$ is obtained.

Step 6: Based on the obtained $\hat{x}(t)$, calculate the wavenumber spectrum and the dissipation rate of TKE (turbulence kinetic energy). Compare the wavenumber spectrum with the standard Nasmyth theoretical spectrum to evaluate the turbulence shear signal denoising effect.

## 4. Result and Analysis

In this work, the EMD-PCA algorithm is used to eliminate the noise of the observed ocean turbulence data, and the standard Nasmyth theoretical spectrum is selected as the standard to evaluate the denoising effectiveness. In the filed test, the instrument collected abundant sea data from the deep sea for a long time at a fixed level. The shear signal sample processed in this paper is 3 min long, and the corresponding velocity U = 0.28 m/s, which represents a 250-m velocity profile.

Turbulence shear signals are measured from two orthogonal shear probes equipped on the turbulence observation system. The two shear probes can measure the velocity shear (*∂w*/*∂z* and *∂v*/*∂z*) in two orthogonal directions, respectively. The output voltage of the shear probe can be expressed as [23]:

(11) $$E_{p}=2\sqrt{2}SUw$$

where U is the velocity of the shear probe, w is the velocity perpendicular to the probe and S is the sensitivity of the shear probe. Based on the Taylor frozen assumption, we can obtain the shear signals [24]:

(12) $$\frac{\partial w}{\partial z}=\frac{1}{U}\frac{\partial w}{\partial t}=\frac{\partial E_{p}/\partial t}{2\sqrt{2}SU^{2}}$$

**Figure 2:**
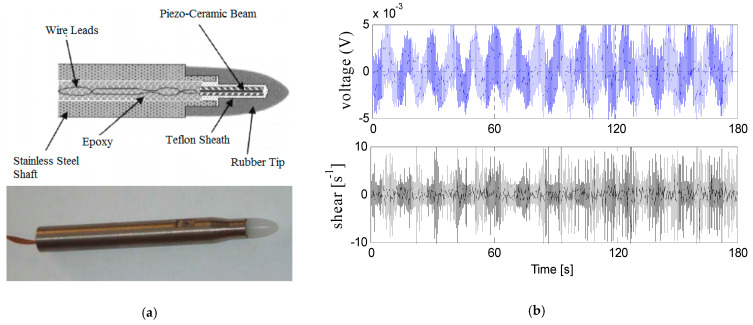
The sensors and the collected ocean turbulence shear signals. (**a**) The structure of the shear probes; (**b**) the original ocean turbulence shear signals.

The unprocessed shear signal sample contains contamination from the instrument motions and vibrations, which obscures the true environmental shear signal in the time domain (shown in Figure 2). The blue wavy lines in Figure 2b represents the raw collected voltage and the black wavy lines represents the turbulence shear calculated using Equations (11) and (12). In Figure 2, the length of the shear signal sample is 180 s. The signals on the top panel are the stream-wise velocity fluctuation (*∂u*/*∂x*, corresponding to shear1) and the signals on the bottom panel are the athwartships component of the velocity fluctuation (*∂v*/*∂x*, in the body frame and corresponding to shear3). Both of the perpendicular components *∂u*/*∂x* and *∂v*/*∂x* have strong fluctuations.

**Figure 3:**
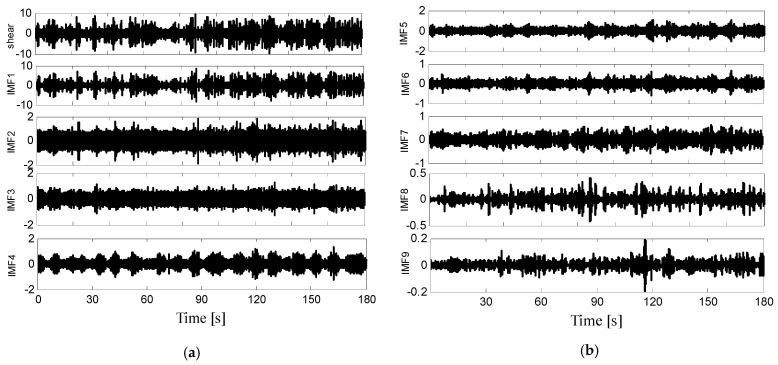
Comparison of the raw velocity shear signals and the IMF modes. (**a**) The raw velocity shear signals and the IMF modes from IMF1 to IMF4; (**b**) The IMF modes from IMF5 to IMF9.

Comparison of the raw velocity shear signals and the decomposed IMF modes is shown in Figure 3. It is easy to find that after the resampling, the resampled signals hold the same detail information as the original signals. Here, we only present one of the two perpendicular velocity components that decomposed using the EMD method. All the IMF modes have different mean frequency and time scales.

**Figure 4:**
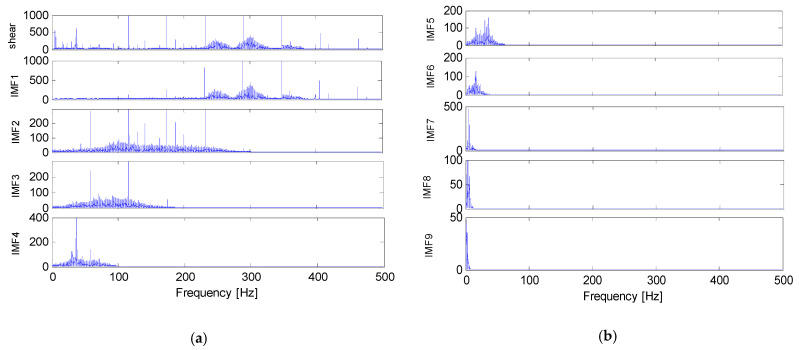
Frequency comparison of the raw velocity shear signals and the IMF modes. (**a**) The frequencies of the raw velocity shear signals and the IMF modes from IMF1 to IMF4; (**b**) The frequencies of IMF modes from IMF5 to IMF9.

In Figure 3, each IMF modes has its own time scales and frequency. IMF1–IMF9 represent the different parts of the original ocean turbulence shear signal with the frequency from high to low. The time scales of the nine IMF modes become larger as the orders increase, while the frequency and amplitudes become smaller. The variation tendency of IMF1 is the most similar to the original shear signals. Frequency comparison of the raw velocity shear signals and the decomposed IMF modes is shown in Figure 4. The horizontal axis represents the frequency and the vertical axis represents the amplitude.

**Figure 5:**
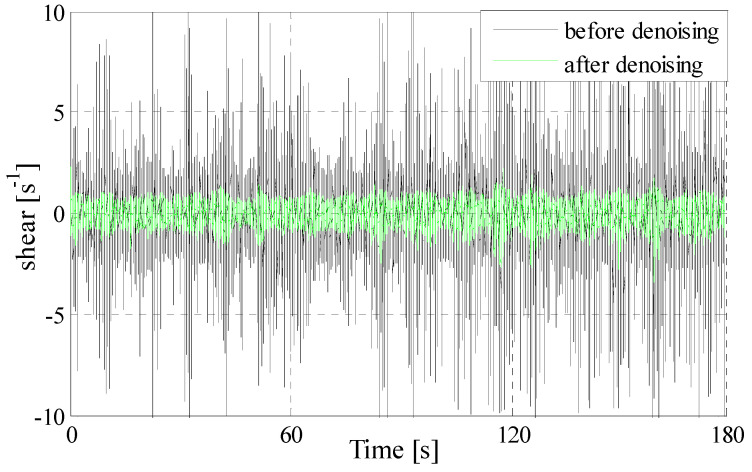
Comparison of signals before and after noise elimination in time domain.

PCA is used to analyze the principal component of the obtained IMF1 and estimate the noise energy. After the noise energy contained in all IMF components are estimated, the appropriate number of principal components is selected for each IMF component. After the denoising of IMF components in each layer is completed, the reserved IMF components and the remainder are accumulated and reconstructed. So, the turbulence shear signal after denoising is obtained. The comparison between the reconstructed turbulence shear signal and the unprocessed one are shown in Figure 5.

To further verify the effectiveness of the improved algorithm based on EMD-PCA, we compare it with the classical cross spectrum algorithm. Signals processed with the EMD-PCA algorithm are transformed into the frequency domain using the Fast Fourier Transform (FFT). Based on the Taylor freezing theorem, the shear frequency power spectrum is converted to wavenumber domain k=f/U and can be compared with the standard Nasmyth theoretical spectrum, which is regarded as a criterion for evaluating the effectiveness of turbulence signals. The dissipation rate of turbulence kinetic energy is calculated with the corrected shear signals using spectral integration [23]:

(13) $$\varepsilon =7.5\nu \bar{{\left(\frac{\partial w}{\partial z}\right)}^{2}}=7.5\nu \int_{k_{\min}}^{k_{\max}}\Phi (k)dk$$

where *∂w*/*∂z* is the vertical shear, Φ(*k*) is the wavenumber spectrum of the shear signals, ν is the coefficient of viscosity and $\nu \approx 1.64\times 10^{-6}m^{2}s^{-1}$.

**Figure 6:**
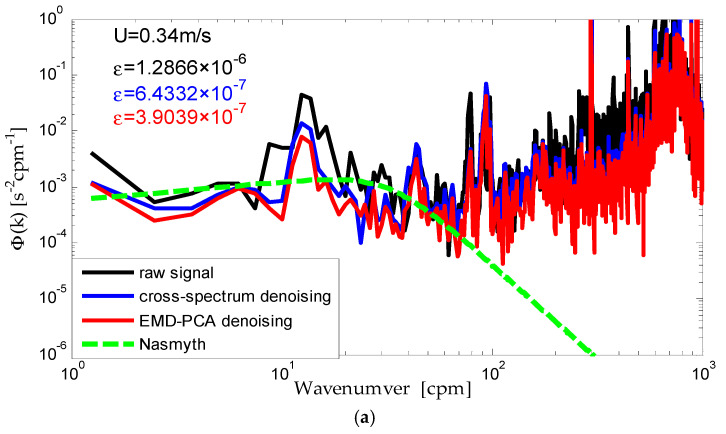

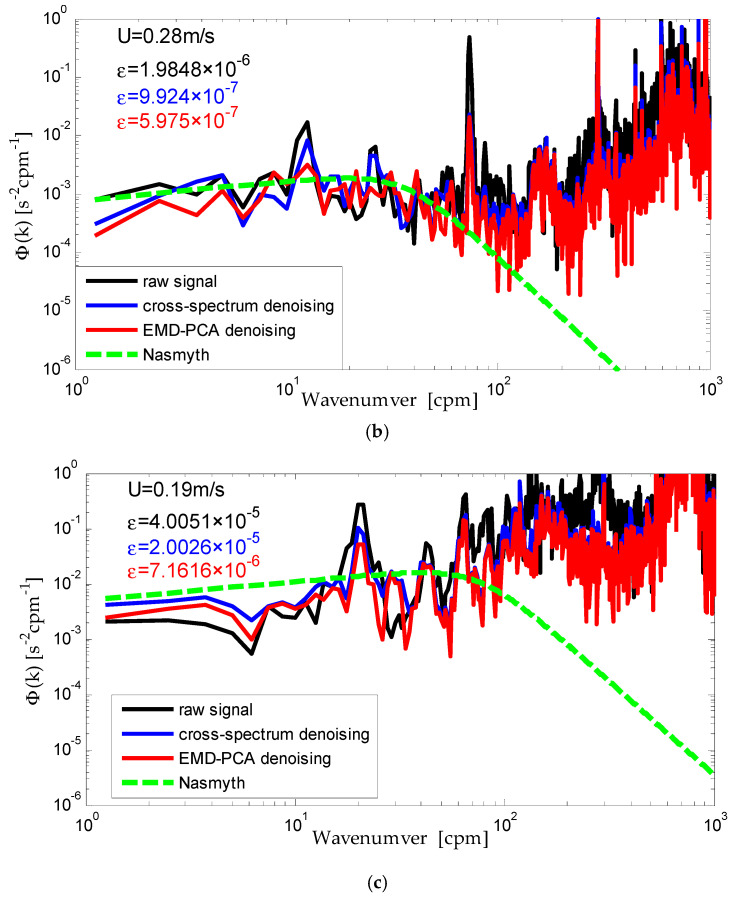
Comparison of denoising effectiveness. (**a**) The corresponding current speed is 0.34 m/s; (**b**) the corresponding current speed is 0.28 m/s; (**c**) the corresponding current speed is 0.19 m/s.

The comparison results are shown in Figure 6. In Figure 6, the three solid curves are the observed spectra, respectively, where the black curve is the raw signal spectrum before denoising, the red one is the spectrum after being corrected with the improved method proposed in this paper and the blue one is the spectrum after being corrected with the cross-spectrum denoising algorithm. The green dashed curve is the Nasmyth theoretical spectrum. The current speed is 0.34 m/s in Figure 6a, 0.28 m/s in Figure 6b and 0.19 m/s in Figure 6c.

It is obvious that compared with the cross-spectrum denoising algorithm, the observed spectrum corrected with the improved denoising algorithm based on EMD-PCA better eliminates the noise spike and the spectrum shape agrees much closer with the Nasmyth theoretical spectrum up to 80 cpm, which means the low wavenumber noise apparent in the raw spectrum is removed more effectively. Furthermore, the dissipation rate of the TKE computed with the corrected shear signals using the improved denoising algorithm drops near an order of magnitude compared to the raw measured data, which provides reliable and effective data for the further analysis of the turbulence characteristics.

**Figure 7:**
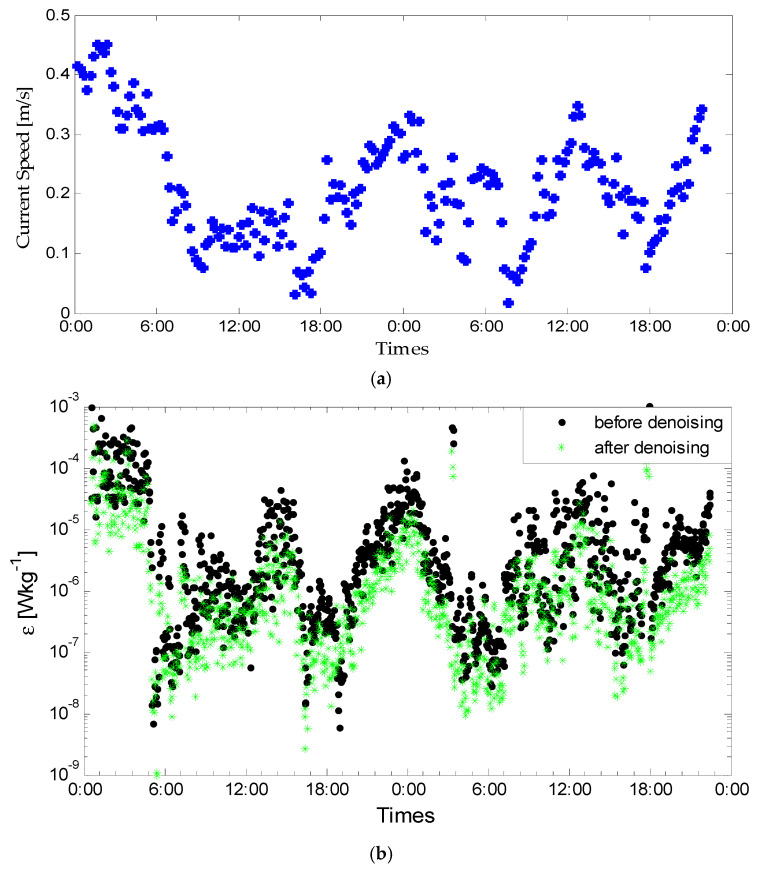
(**a**) The variation of two days of current velocity; (**b**) comparison of calculated dissipation rate of TKE calculated from the corresponding two days turbulence shear.

In addition, the improved denoising algorithm based on EMD-PCA is also performed on two days of turbulent velocity shear signals. Here, we also add the variation of the corresponding measured current velocity, shown in Figure 7a. The dissipation rate of TKE before and after denoising are calculated and compared, which are shown in Figure 7b. The variation curve of the turbulence kinetic energy dissipation rate represents the semi-diurnal tide characteristic. When the macroscopic parameters fluctuate with the tide period, the instantaneous local flow characteristics, as well as the turbulent structure, will be affected by a variety of mechanisms.

In addition, this work also adopts two criteria to evaluate the denoising effectiveness of the improved denoising algorithm based on EMD-PCA: MSE (mean square error) and SNR (signal-to-noise ratio). MSE is always used to evaluate the change degree of the data and the smaller of MSE, the better of the algorithm performance. SNR represents the ratio between signal and noise and the higher of SNR, the less noise it contains and the better of the algorithm efficiency [25,26].

(14) $$MSE=\sqrt{\frac{1}{N}({\sum_{n=1}^{N}\left(x(t)-\hat{x}(t)\right)}^{2})}$$

(15) $$SNR=10\times \log_{10}\left[\sum_{n=0}^{N-1}\frac{x(t)}{{\left(x(t)-\hat{x}(t)\right)}^{2}}\right]$$

In this work, the turbulent velocity shear signals that collected at different current speed are processed using the improved denoising algorithm based on EMD-PCA. The calculated results of MSE and SNR at different current speed are shown in Table 1.

It can be seen that at different current speed, the values of MSE calculated from the original signal and the denoised signal is relatively small; meanwhile, the SNR is relatively high, which can verify the good denoising effectiveness.

## 5. Conclusions

Ocean turbulence collected signals are the basis to analyze the turbulence mixing characteristics. In this work, aiming at eliminating the noise component in the collected turbulence signals, an improved turbulence denoising method combining the EMD and PCA is proposed. First, the raw turbulence signals are decomposed using EMD method and noise energy of each IMF is estimated. Second, the PCA method is implemented on each IMF to select the appropriate number of principal components. Finally, the denoised signal is reconstructed based on the selected effective IMF. Ocean turbulence shear signals collected in the South China Sea with a moored turbulence observation instrument are used to evaluate the performance of the improved denoising method. Samples under different current speed are chosen. By comparing with the cross-spectrum denoising method, the results show that the improved method can effectively and reliably eliminate the vibration noise. Two days of dissipation rates of TKE before and after denoising are also calculated and compared, which proved that the improved method can well eliminate the noise and can supply effective turbulence signals for further analysis of ocean turbulence.

## Figures and Tables

**Table 1:** Results of MSE and SNR at different velocities.

Current Speed (m/s)	MSE	SNR
0.26	0.102	34.52
0.31	0.087	30.16
0.35	0.093	36.72

## Data Availability

Not applicable.

## Abbreviations

EMD	Empirical Mode Decomposition
PCA	Principle Component Analysis
IMF	Intrinsic Mode Function
SCS	South China Sea
TKE	Turbulent Kinetic Energy
MTMI	Moored Turbulence Measuring Instrument
OUC	Ocean University of China
FFT	Fast Fourier Transform
MSE	Mean Square Error
SNR	Signal-to-Noise Ratio
